# 

**Published:** 1871-05

**Authors:** 


### CONTENTS.

COMMUNICATIONS.

First Dentition ... 189
Dental Education ... 199
Causes Retarding Dental Progress ... 204
Vital Phenomena ... 214
Alleged Cause of Death from Nitrous Oxyd ... 224

PROCEEDINGS OF SOCIETIES.

Proceedings of the Twenty-seventh Annual Meeting of the Mississippi Valley Dental Association, March 1st, 1871 ... 226
Preserv [illegible]ion of the Cadaver ... 229

EDITORIAL.

Filling Teeth ... 230
Nitrous Oxyd Rumors ... 232
To the Point ... 234
Death of a Noted Southern Physician ... 234

